# A Cross-Sectional Observational Study on the Coexistence of Erectile Dysfunction and Premature Ejaculation

**DOI:** 10.1016/j.esxm.2021.100438

**Published:** 2021-09-24

**Authors:** Chieh‑Wen Chin, Chia Mu Tsai, Jen-Tai Lin, Yin-Shen Chen, I-Hsuan Chen, Bang-Ping Jiann

**Affiliations:** 1 Division of Urology, Department of Surgery, Kaohsiung Veterans General Hospital, Kaohsiung City, Taiwan; 2 Division of Urology, Department of Surgery, Kaohsiung Veterans General Hospital, Kaohsiung; School of Medicine, National Yang-Ming University, Taipei; and College of Health and Nursing, Meiho University, Pingtung, Taiwan

**Keywords:** Erectile Dysfunction, Premature Ejaculation, Coexist, Temporal Relationship

## Abstract

**Introduction:**

The interplay between erectile dysfunction (ED) and premature ejaculation (PE) needs more studies to clarify.

**Aim:**

To evaluate the risk factors and temporal relationship for the coexistence of ED and PE.

**Methods:**

The data were derived from clinical history.

**Main Outcome Measure:**

The diagnosis of ED and PE was based on self-report and validated questionnaires.

**Results:**

Based on the chief complaint, 1,893 participants were recruited as ED group and 483 participants as PE group from 2014 to 2020. One third of ED and PE groups reported comorbid PE and ED. Of the ED group, 4.1% (n = 77) had lifelong PE, 18.0% (n = 341) had acquired PE and 9.7% (n = 184) had subjective or natural variable PE. Of the PE group, ED was reported in 22.0% (n = 40) of lifelong PE, 33.9% (n = 65) of acquired PE, and 37.6% (n = 41) of subjective or natural variable PE, *P* < .01. With adjustment of potential confounders, the ED severity was associated with increased risk of acquired PE, while acquired PE was associated with higher risk of ED than lifelong PE. In cases of comorbid lifelong PE and ED (n = 117), 22.2% reported the onset of both dysfunctions being about the same time, whereas 77.8% reported ED occurred behind PE with an average 23.3 years lag. In cases of comorbid acquired PE and ED (n = 406), 52.2% reported the onset of both dysfunctions being about the same time, 34.2% reported ED happened behind PE and 13.5% reported PE emerged behind ED.

**Conclusion:**

Organic pathogenesis was least likely to be responsible for the link between PE and ED. When acquired PE and ED coexist, treating ED first or concomitantly according to their temporal order is an appropriate management algorithm.

**Chieh‑Wen Chin, Chia Mu Tsai, Jen-Tai Lin, et al. A Cross-Sectional Observational Study on the Coexistence of Erectile Dysfunction and Premature Ejaculation. Sex Med 2021;9:100438.**

## INTRODUCTION

Erectile dysfunction (ED) and premature ejaculation (PE) are considered two distinct sexual dysfunctions with each own diagnostic criteria and are most frequent self-reported sexual concerns in men.^1^ Epidemiological studies demonstrated that ED and PE often coexist. Twenty-three percent of men with ED in a Swedish nationally representative sample reported to have an early ejaculation problem.^2^ PE patients had a significantly increased risk of ED with an odds ratio (OR) of 3.68 reported in a meta-analysis.^3^ Variation in study methods including the criteria for defining sexual dysfunctions and study population yielded discrepancy in the magnitude of the association between ED and PE.^3^ The definition of PE has gone through evolution in the past decade when more strict diagnostic criteria of PE was initiated by the Diagnostic and Statistical Manual of Mental Disorders, Fifth Edition (DSM-5) in 2013^4^ and the International Society for Sexual Medicine (ISSM) in 2014,^5^ and the most up to date by the American Urological Association (AUA) guideline in 2020.^6^ The new criteria of PE has never been applied in clinical studies to investigate the coexistence of ED and PE.

Several hypotheses were proposed for the coexistence of ED and PE. PE patients may attempt to reduce their sexual excitement to delay ejaculation and thus be superimposed with ED.^7^ They may report to have ED due to an early detumescence after ejaculation.^8^ Erectile dyfunction patients may need intense stimulation to attain and maintain penile erection which leads to PE.^7^ Besides, psychosocial distress associated with ED or PE contribute to their coexistence.^9^^,^^10^ However, scanty scientific evidence was available to support these hypotheses. Recognizing the temporal relationship between sexual dysfunctions may shed lights on their interplay but, to our knowledge, such data has never been reported before. Furthermore, the link between PE and ED was infrequently analyzed according to the types of PE.^3^

In addition to the above explanation for the link between PE and ED, is there any organic pathogenesis for their comorbidity? Herein, diagnostic criteria for PE conforming to the ISSM definition^5^ were applied to assess the co-occurrence of PE and ED in a cohort of outpatients presenting with a chief complaint of ED or PE.

## AIM

The aim of this study was to evaluate the coexistence of ED and PE and its risk factors and temporal relationship.

## METHODS

### Study Participants

This was a cross-sectional observational study. The study population was recruited from consecutive cases presenting with a chief complaint of ED or PE for the first time in the outpatient department (OPD) of two hospitals from April 2014 to November 2020. Subjects with a history of prostate cancer, spinal cord injury, pelvic trauma, advanced malignancy, radical pelvic surgery, illicit drug abuse, or alcoholism would be excluded.

The medical history was enquired by the question of “*Have you been diagnosed to have diabetes mellitus (DM), hypertension (HT), dyslipidemia, or major cardiovascular events (MACE)*?” Blood pressure, body weight and height, and waist circumference were measured by a nurse. The body mass index (BMI) was derived from the body weight and height (kg/m^2^). Overweight was defined as BMI ≥ 24 and < 27 kg/m^2^ and obesity as ≥ 27 kg/m^2^. Laboratory tests including fasting blood sugar, lipid profile, and total testosterone would be arranged, if such data is not available in the past one year. Dyslipidemia was defined as having a self-reported dyslipidemia or serum level of total cholesterol ≥ 240 mg/dL, low-density lipoprotein cholesterol ≥ 160 mg/dL, high-density lipoprotein cholesterol < 40 mg/dL or triglyceride ≥ 200 mg/dL. DM was defined as having a self-reported DM, fasting plasma glucose ≥ 126 mg/dL or HbA1c ≥ 6.5%. HT was defined as having a self-reported HT. Hypogonadism was defined as a plasma total testosterone level below 348 ng/dL.

Subjects with a chief complaint of ED would be routinely screened for comorbid PE by a global assessment question (GAQ) of “*Do you suffer from premature ejaculation in most of your sexual attempts*?” Subjects with a chief complaint of PE would be routinely screened for comorbid ED by a GAQ of “*Do you have erectile dysfunction*?” All participants were asked to complete the Sexual Health Inventory for Men (SHIM)^11^ and Premature Ejaculation Diagnostic Tool (PEDT).^12^ The intravaginal ejaculation latency time (IELT) was determined by self-estimate.

The study protocol was reviewed and approved by the Institutional Review Board (IRB) at the institution. A waiver of written informed consent was approved by the IRB.

### Outcome Measures

The diagnosis of ED relied on self-reporting (having a chief complaint of ED or a positive response to the ED screening GAQ). The severity of ED was determined by the total SHIM score as severe ED (1–7), moderate ED (8–11), mild to moderate ED (12–16), mild ED (17–21), and no ED (22–25).^11^

The ISSM definition of lifelong and acquired PE has three key elements: control, bother and IELT.^5^ A PEDT score ≥ 11^12^ was used as a surrogate for the criteria regarding control and bother. Accordingly, the participants who had a chief complaint of PE or a positive response to the PE screening GAQ were classified into the following three subgroups. Lifelong PE was defined as the problem starting from the first sexual experience, a PEDT score ≥ 11^12^ and an IELT ≤ 1 min.^5^ Acquired PE was defined as the problem happening after a period of normal ejaculation, a PEDT score ≥ 11^12^ and an IELT ≤ 3 min.^5^ Subjects who had the problem but did not fulfill the diagnostic criteria for lifelong or acquired PE were classified as having subjective or natural variable PE conforming to the provisional diagnosis proposed by Marcel Waldinger.^13^

The duration of the sexual problem was determined by the subject's recall. If the answer was a range, a mean value of the range would be adopted. The temporal order between the concomitant ED and PE was derived from the duration of each problem and ascertained by the participant.

### Statistical Methods

The mean ± standard deviation (range) or percentage (number) were used to summarize the descriptive data when appropriate. The Chi-Squared test was used for comparison of categorical variables. One-way analysis of variance was assessed for continuous variables and Scheffé test was used for post-hoc analysis of multiple comparisons. Multiple logistic regression was used to estimate the relative odds of having a sexual problem, given exposure to the variable of interest with adjustment of potential confounders as appropriate.

Data entry was performed using Excel 2019 (Microsoft, Redmond, W.A., USA). Statistical analyses were executed using IBM SPSS Statistics for Windows, version 26 (IBM Corp., Armonk, N.Y., USA). The null hypothesis was rejected when a *P* value was less than .05.

## RESULTS

**Figure 1:**
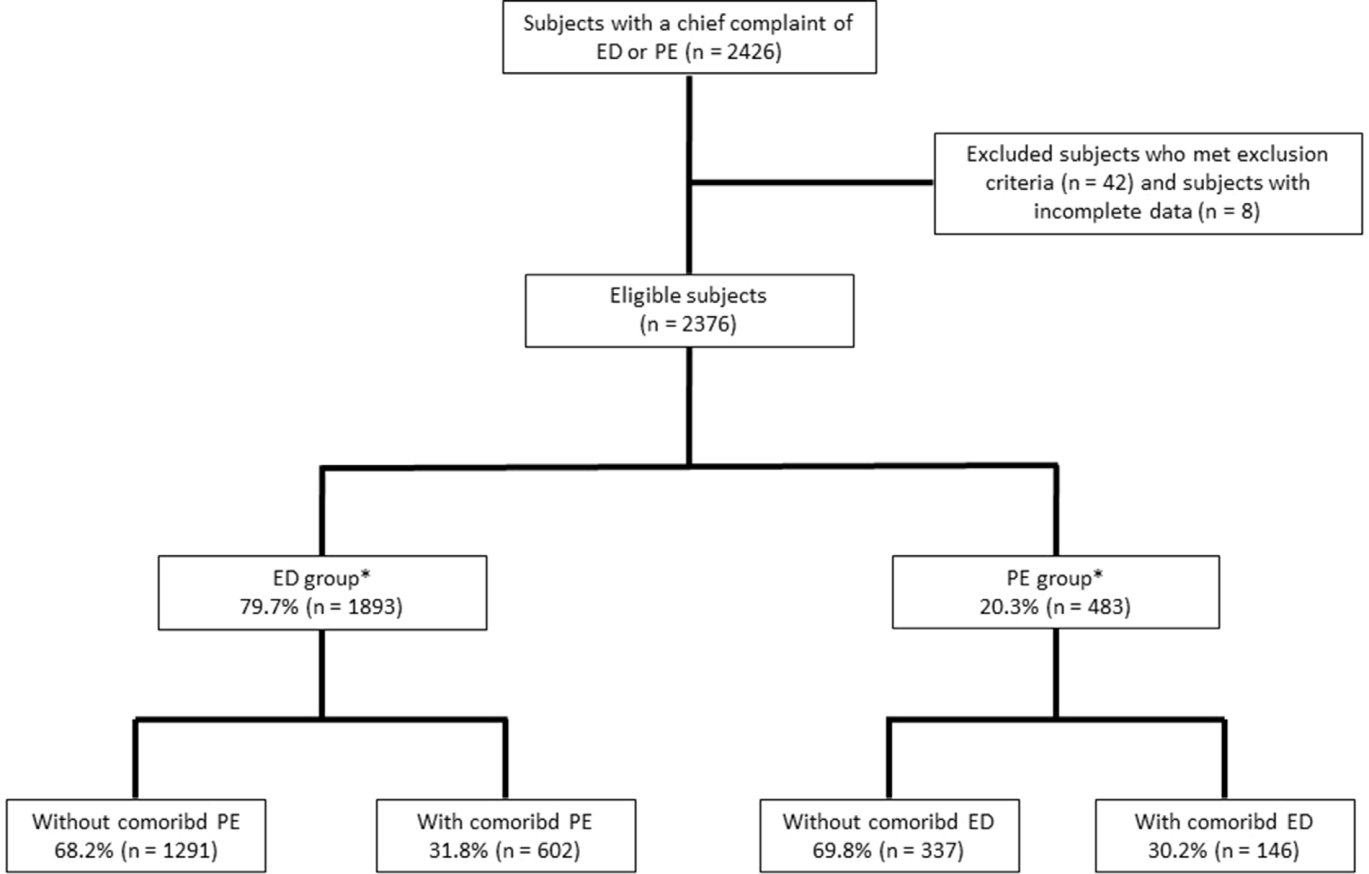
Flow diagram of study participants Abbreviations: ED = erectile dysfunction, PE = premature ejaculation. *Classification of ED and PE groups was based on the subject's chief complaint .

During the study period, a total of 2426 patients visited the OPD with a chief complaint of ED or PE for the first time. After excluding 42 subjects who met the exclusion criteria and 8 subjects who had incomplete SHIM or PEDT score, 2376 subjects were eligible for analysis (Figure 1). According to the chief complaint, 79.7% (n = 1893) of them were categorized as the ED group and 20.3% (n = 483) as the PE group. The PE group was younger in age than the ED group (43.8 ± 12.2《20–76》years. vs 53.2 ± 12.7《20–88》years, *P* < .001).

### Comorbid PE in the ED Group

Based on the SHIM score, the distribution of ED severity of the ED group was no ED (3.2%), mild ED (12.4%), mild to moderate ED (26.6%), moderate ED (23.8%), and severe ED (34.1%). Of the ED group, 31.8% (n = 602) had comorbid PE with lifelong PE in 4.1% (n = 77), acquired PE in 18.0% (n = 341), and subjective or natural variable PE in 9.7% (n = 184).

Table 1 compares the characteristics among four subgroups of the ED group: ED without PE and ED with lifelong, acquired, or subjective or natural variable PE. Among the subgroups, significant differences existed in their age, DM prevalence, ED duration, total SHIM score and ED severity, whereas no significant difference was found in their prevalence of obesity, HT, dyslipidemia, MACE and hypogonadism.

**Table 1:** Comparisons between the subjects without and with different types of comorbid PE in the ED group

Variables	Subjects without PE (n = 1291)	Subjects with lifelong PE (n = 77)	Subjects with acquired PE (n = 341)	Subjects with Probable PE (n = 184)	*P* value
Age, y	54.1 ± 13 (20–88)	51.2 ± 12.8 (25–70)	51 ± 11.5 (20–75)	52.3 ± 12 (24–75)	*P* <.001
Obesity* (BMI ≥ 27 kg/m^2^)	30.1% (389)	23.4% (18)	27.6% (95)	27.7% (51)	No significance
Diabetes mellitus	29.4% (379)	23.4% (18)	17.6% (60)	15.2% (28)	*P* <.001
Hypertension	33.0% (426)	28.6% (22)	27.6% (94)	31.0% (57)	No significance
Dyslipidemia	50.6% (653)	53.2% (41)	44.9% (154)	47.8% (88)	No significance
MACE	7.2% (93)	9.1% (7)	5.9% (20)	8.2% (15)	No significance
Hypogonadism*	27.4% (283)	23.3% (14)	23.8% (62)	22.6% (33)	No significance
ED duration, y	2.7 ± 3.8 (0.3–40)	3.9 ± 5.2 (0.3–30)	3.2 ± 4.1 (0.3–30)	2.8 ± 3.7 (0.3–30)	*P* <.05
Total SHIM score	10.7 ± 5.9 (1–25)	9.3 ± 4.9 (1–23)	9.5 ± 4.9 (1–25)	11.6 ± 5.6 (1–23)	*P* <.001
ED severity					
*Normal*	3.8% (49)	1.3% (1)	0.9% (3)	3.8% (7)	*P* <.001
*Mild*	13.5% (174)	6.5% (5)	7.6% (26)	15.8% (29)	
*Mild to moderate*	26.6% (344)	24.7% (19)	24.0% (82)	32.1% (59)	
*Moderate*	22.2% (286)	28.6% (22)	29.2% (99)	23.4% (43)	
*Severe*	33.9% (438)	39.0% (30)	38.4% (131)	25.0% (46)	

Abbreviations: BMI = body mass index, ED = erectile dysfunction, MACE = major adverse cardiovascular events, PE = premature ejaculation, SHIM = Sexual Health Inventory for Men,

⁎ Hypogonadism is defined as a serum total testosterone level below 348 ng/dL.

A multiple logistic regression with independent variables of age groups, obesity, DM, HT, dyslipidemia, and ED severity and duration was carried out to estimate their ORs for comorbid non-typing PE. The age group of ≥ 70 years (OR = 0.21, *P* < .001; reference: 20–29 yrs), DM (OR =.49, *P* < .001), ED severity (OR = 1.49, 1.89 and 1.76 for mild to moderate, moderate and severe ED, respectively, all *P* < .05; reference: no ED) and ED duration (OR = 1.03, *P* <.01) were significantly related with comorbid non-typing PE. Another two multiple logistic regressions with the same independent variables were applied to estimate theirs ORs for lifelong and for acquired PE. Lifelong PE was related to obesity (OR = 0.56, *P* < .05) and ED duration (OR = 1.05, *P* < .05). Acquired PE was associated with age group of ≥ 70 yrs (OR = 0.28, *P* < .05), overweight (OR =.74, *P* < .05), DM (OR = .48, *P* < .001) and ED severity (OR = 1.92, 2.95 and 2.96 for mild to moderate, moderate and severe ED, respectively, all *P* < .01).

### Comorbid ED in the PE Group

Of the PE group (n = 483), 37.7% (n = 182) were classified as lifelong, 39.8% (n = 192) as acquired, and 22.6% (n = 109) as subjective or natural variable PE with a mean age of 42.8 ± 12.7 (20–76) years, 44.4 ± 11.8 (20–74) years and 44.3 ± 12.2 (20–70) years, respectively, *P* = .39. Of them, 30.2% (n = 146) had comorbid ED by the GAQ, whereas 85.5% (n = 413) would be classified as having ED by the SHIM score. Comorbid ED was reported in 22.0% (n = 40) of lifelong PE, 33.9% (n = 65) of acquired PE and 37.6% (n = 41) of subjective or natural variable PE, *P* < .001.

Table 2 compared the characteristics among the PE group stratified by the presence of comorbid ED. Subjects with comorbid ED were older in age and had more DM, HT, dyslipidemia, MACE and longer PE duration than those without. No significant difference existed in their PEDT sores, IELT and the prevalence of obesity and hypogonadism. There was still no significant difference in the PEDT scores and IELT between those with and without ED after stratifying the types of PE. A multiple logistic regression with independent variables of age groups, obesity, DM, HT, dyslipidemia, types of PE and PE duration was performed to estimate their ORs for comorbid ED. Age groups of 60–69 and ≥ 70 yrs, (OR = 2.64 and 7.99, respectively, both *P* < .05; reference: 20–29 yrs), DM (OR = 2.35, *P* < .05), dyslipidemia (OR = 3.20, *P* < .001), and subgroups of acquired and subjective or natural variable PE (OR = 1.85 and 2.15, respectively, both *P* < .05; reference: lifelong PE) were significantly associated with comorbid ED in the PE group.

**Table 2:** Comparisons between subjects with and without comorbid ED in the PE group

Variables	Subjects without comorbid ED (n = 337)	Subjects with comorbid ED (n = 146)	*P* value
Age, y	41.5 ± 11.4 (20–76)	48.9 ± 12.6 (20–76)	*P* <.001
Obesity (BMI ≥ 27 kg/m^2^)	21.7% (73)	29.5% (43)	No significance
Diabetes mellitus	4.2% (14)	17.8% (26)	*P* <.001
Hypertension	8.6% (29)	21.2% (31)	*P* <.001
Dyslipidemia	23.7% (80)	52.7% (77)	*P* <.001
MACE	0.3% (1)	3.4% (5)	*P* <.01
Hypogonadism*	19.9% (39)	27.5% (30)	No significance
PE duration, y	9.9 ± 10.4 (0–50)	12.3 ± 13.2 (0.3–50)	*P* <.05
Total PEDT score	15.1 ± 3.6 (3.0–20.0)	15.1 ± 3.8 (4.0–20.0)	No significance
PE types			
*Lifelong PE*	42.1% (142)	27.4% (40)	*P* <.01
*Acquired PE*	37.7% (127)	44.5% (65)	
*Probable PE*	20.2% (68)	28.1% (41)	
IELT, sec	72.1 ± 68.3 (0–600.0)	77.2 ± 64 (3.0–300.0)	No significance

Abbreviations: BMI = body mass index, ED = erectile dysfunction, IELT = Intravaginal ejaculatory latency time, MACE = major adverse cardiovascular events, PE = premature ejaculation, PEDT = Premature Ejaculation Diagnostic Tool

⁎ Hypogonadism is defined as a serum total testosterone level below 348 ng/dL.

## Temporal Relationship of Concomitant PE and ED

**Figure 2:**
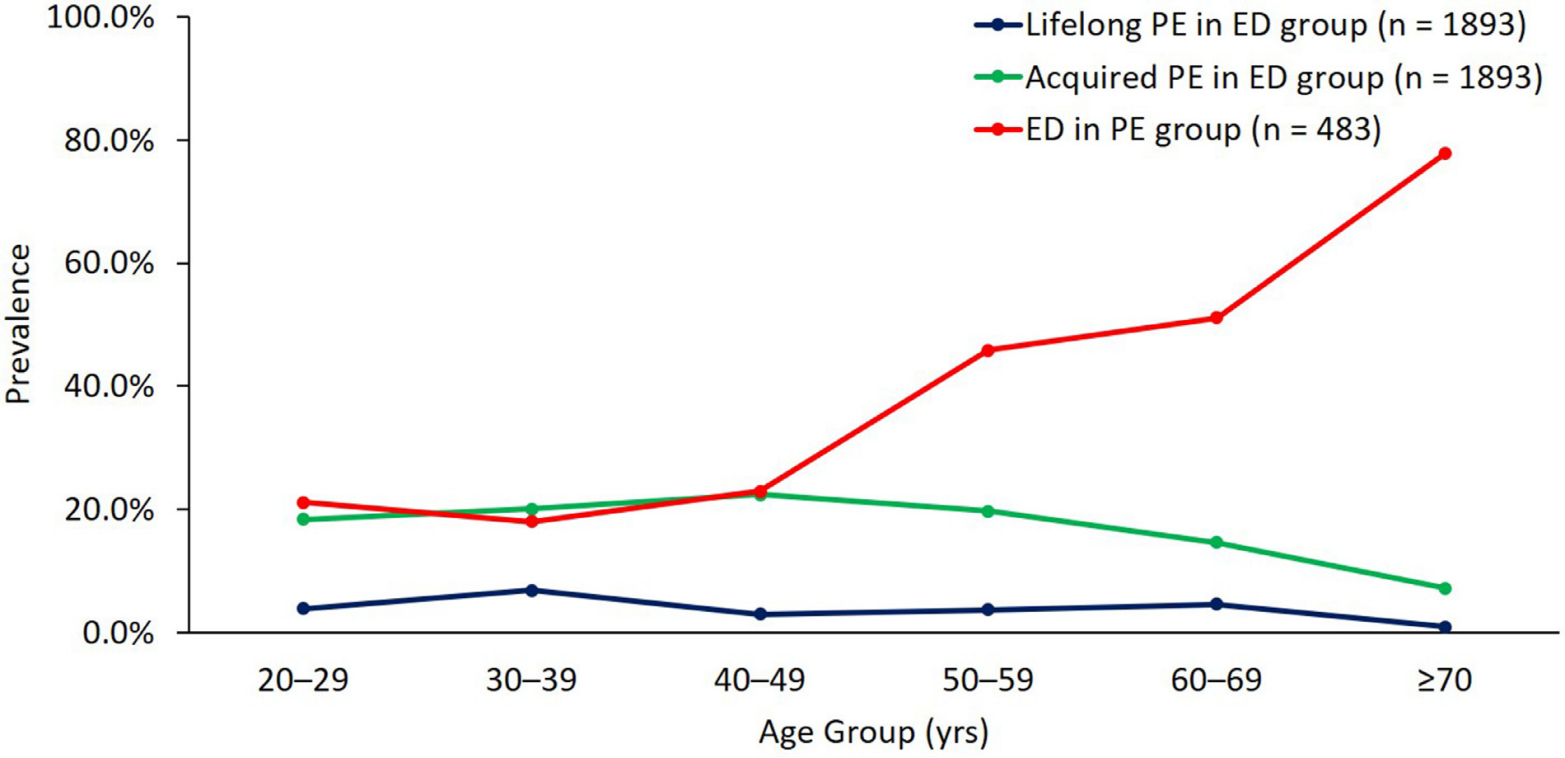
The prevalence of lifelong and acquired PE in the ED group (n = 1893) and ED in the PE group (n = 483) stratified by age groups Abbreviations: ED = erectile dysfunciton, PE = premature ejaculation.

The prevalence of lifelong and acquired PE in the ED group and ED in the PE group stratified by age group is shown in Figure 2**.** The prevalence of lifelong PE in the ED group fluctuated slightly before the age of 70 years and dipped after then, while the prevalence of acquired PE dipped after the age of 60 yrs. The prevalence of ED in the PE group kept steady before the age of 50 years, then increased steeply after then.

**Figure 3:**
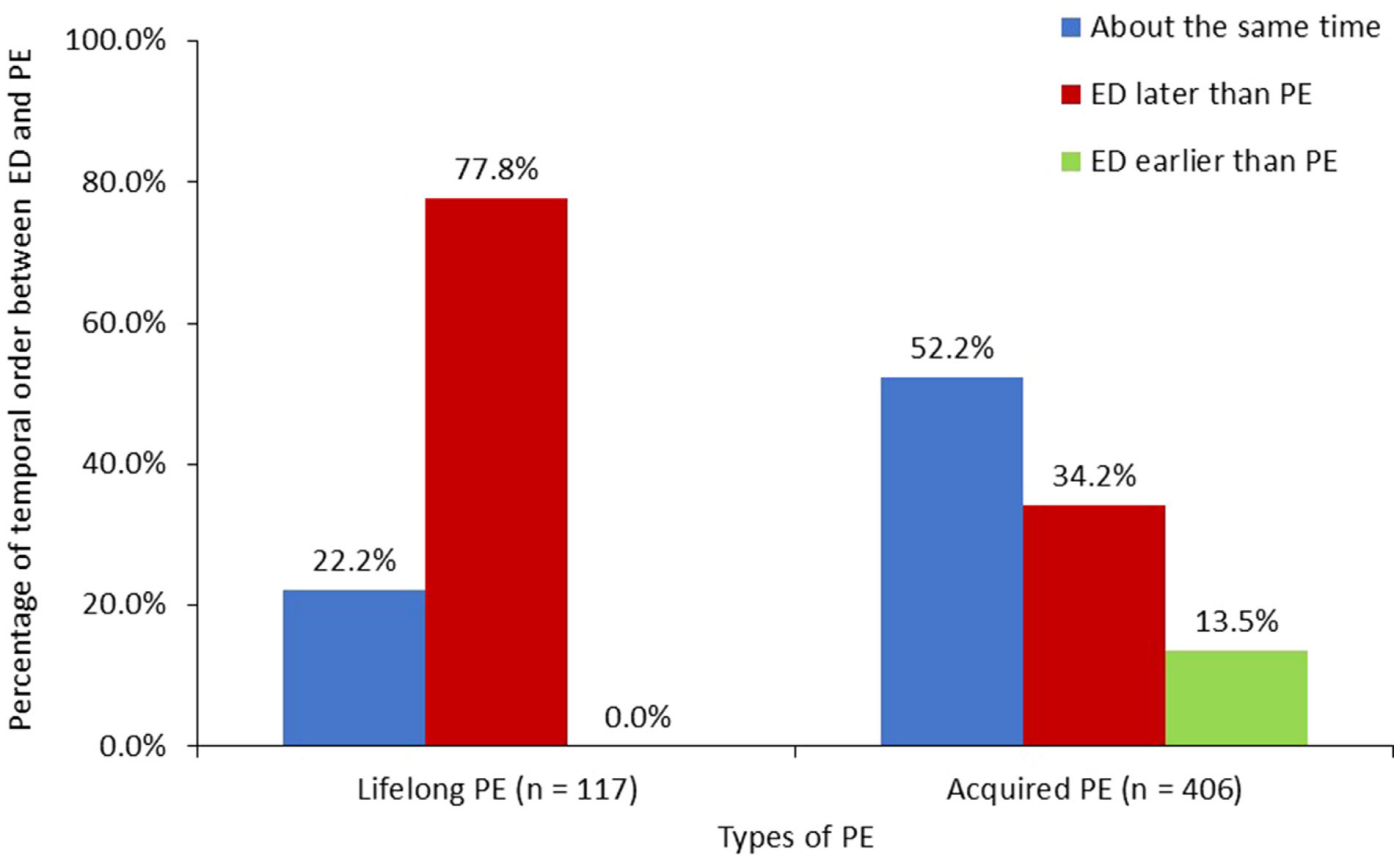
The temporal relationship of the onset time of concomitant PE and ED among those with lifelong and those with acquired PE Abbreviations: ED = erectile dysfunction, PE = premature ejaculation.

In subjects with concomitant PE and ED, both the time lag and temporal order of the two dysfunctions displayed no significant difference between the ED and PE groups after stratifying PE types, whereas significant difference existed in these variables between different types of PE. Figure 3 compares the temporal relationship of concomitant PE and ED among subjects with lifelong PE and those with acquired PE. Of those with lifelong PE, 77.8% (91/117) reported the onset of ED behind that of PE with a mean time lag of 23.3 ± 13.0 (median 24, range 0.1–49.7) years and 22.2% (26/117) reported the onset being about the same time. Of those with acquired PE, 52.2% (212/406) reported the onset of both dysfunctions as about the same time, 34.2% (139/406) reported the onset of ED behind that of PE with a mean time lag of 6.2 ± 7.9 (median 3, range 0.1–45.5) years and 13.5% (55/406) reported the onset of PE behind that of ED with a mean time lag of 2.7 ± 3.1 (median 2, range 0.1–17) years.

## DISCUSSION

Of this setting, the number of patients seeking help for the ED problem was four times that for the PE problem. About one third of both the ED and PE groups had a concomitant condition of each other. Of the ED group, the prevalence of lifelong PE was 4.1% and acquired PE was 18.0%, which were much higher than the 2% and 4% for their counterparts in the general population.^14^^,^^15^ Of the PE group, the prevalence of self-reported ED was 30.2%, which was three times of the 11.5% prevalence in a Taiwanese sample with a similar age.^16^ Porst et al also reported that the prevalence of ED in men with PE was higher than in those without PE (31.9% vs 11.8%).^17^ These data suggest ED and PE predispose to each other.

In the ED group, ED duration was associated with having lifelong PE and ED severity was associated with having acquired PE. Two studies also reported the association of PE with ED severity but the types of PE were not specified.^18^^,^^19^ Figure 3 showed that 22.2% of the patients with concomitant lifelong PE and ED had both dysfunctions concurrently, which indicated they had onset of ED at a relatively young age and elucidated why an increased ED duration was associated with risk of lifelong PE. Obesity was associated with a less risk of comorbid lifelong PE, which was reported before.^20^ Personality traits such as neuroticism of PE sufferers^21^ may contribute to having less obesity. The age group of ≥ 70 years and DM were found to be associated with a reduced risk for comorbid acquired PE. A large-scale Italian survey reported men with DM or with age ≥ 70 years had less risk for PE.^22^ Ageing or having DM-associated neuropathy might decrease their ability to enhance sexual stimulation and reduce PE risk. In the PE group, in addition to the well-known risk factors for ED, acquired PE increased the risk of comorbid ED when compared with lifelong PE, which was consistent with the findings in a review.^3^

The pattern of temporal relationship of concomitant PE and ED exhibited a significant difference between those with lifelong PE and with acquired PE. The majority (77.8%) of those with concomitant lifelong PE and ED and one third of those with concomitant acquired PE and ED reported the onset of ED behind PE with an average of two decades and six years lag, respectively. The long lag time favored an independent development of the dysfunctions and the formation of ED was attributed to aging or new development of comorbidity. Another 13.5% of those with concomitant acquired PE and ED reported their PE onset behind ED with an average time lag of 2.7 years, suggesting either that the PE be secondary to ED or that they occur independently.

Approximately half (52.2%) of those with concomitant acquired PE and ED and one quarter (22.2%) of those with concomitant lifelong PE and ED reported about the same time for the onset of ED and PE. The indistinguishable onset time suggested that their ED and PE should be seen in a dimensional perspective^3^ and one problem be secondary to the other. In cases of concomitant lifelong PE and ED, the ED was deemed secondary to PE because the subjects were too young to develop primary ED. In cases of concomitant acquired PE and ED, the probability of ED leading to acquired PE was estimated to be approximately three times that of acquired PE leading to ED if acquired PE and lifelong PE have an equal effect on leading to ED. This speculation partially unraveled why 60% (27/45) of men with PE and comorbid ED reported a reduction in severity of PE after treatment with sildenafil.^23^

Lifelong PE was more neurobiological determined, while subjective or natural variable PE was not considered as a manifestation of true medical pathology.^13^ However, lifelong PE was not associated with more comorbid ED than acquired PE and subjective or natural variable PE. Taken together with the results of risk factors and the pattern of temporal relationship of concomitant ED and PE, the link between PE and ED was considered to be least likely through organic pathogenesis. Further research on the temporal relationship between comorbid PE and ED is needed to verify the above conclusion.

### Limitations

Several limitations existed in this study. All the study participants came from outpatients of one principal investigator. Selection bias might exist and caution should be taken in generalizing the results. Psychosocial and relationship factors were not assessed. The IELT was self-estimated without precise timing. Recall bias regarding the onset time of sexual dysfunctions might arise. A substantial part of participants missed the laboratory test that might weaken their associations with ED because of underestimate. The association of HT with ED might be underrated because the diagnosis of HT was based merely on self-report in spite of blood pressure having been measured at the OPD.

The SHIM has been validated for screening ED in the general population but not in specific populations, such as PE sufferers. McMahon reported a 33.3% false positive rate in diagnosing ED in PE sufferers by the SHIM score.^24^ This prompted us to use self-reported ED by a GAQ as well as clinical history to define an ED case. The IELT, PEDT and clinical history were used to define PE, which conformed to the ISSM definition of PE.^5^ Given the current study started in 2014, the updated definition by the 2020 AUA guideline, which expands the IELT criteria as within 2 minutes for lifelong PE and with marked reduction from prior sexual experience for acquired PE,^6^ was not adopted. Strengths of this study also included stratifying types of PE in analysis and assessing the temporal relationship of sexual dysfunctions.

## CONCLUSIONS

Premature ejaculation and ED predispose to each other, meanwhile they may occur independently, especially in men with lifelong PE. An organic pathogenesis was least likely to be responsible for the link between PE and ED. Acquired PE was more associated with ED than lifelong PE. When acquired PE and ED coexist, treating ED first or concomitantly according to their temporal order is an appropriate management algorithm.

## STATEMENT OF AUTHORSHIP

Category 1

Bang-Ping Jiann, Jen-Tai Lin: Conception and Design; Bang-Ping Jiann, Yin-Shen Chen: Acquisition of Data;

Chia Mu Tsai, I-Hsuan Chen: Analysis of Interpretation of Data.

Category 2

Chieh‑Wen Chin: Drafting the Article; I-Hsuan Chen: Revising It for Intellectual Content.

Category 3

Bang-Ping Jiann, Jen-Tai Lin: Final Approval of the Completed Article.
